# Concurrent waterlogging and anthracnose-twister disease in rainy-season onions (*Allium cepa*): Impact and management

**DOI:** 10.3389/fmicb.2022.1063472

**Published:** 2022-12-08

**Authors:** Vanita Navnath Salunkhe, Pranjali Gedam, Aliza Pradhan, Bhaskar Gaikwad, Rajiv Kale, Suresh Gawande

**Affiliations:** ^1^Division of Crop Protection, Indian Council of Agricultural Research (ICAR)-Directorate of Onion and Garlic Research, Pune, Maharashtra, India; ^2^School of Soil Stress Management, Indian Council of Agricultural Research (ICAR)-National Institute of Abiotic Stress Management, Baramati, Maharashtra, India

**Keywords:** anthracnose, biotic stress, concurrent stresses, onion, twister disease, waterlogging

## Abstract

Waterlogging and anthracnose-twister disease are significant obstacles in rainy-season onion cultivation. As a shallow-rooted crop, onions are highly sensitive to waterlogging. Wherever rainy-season onion cultivation has been undertaken, the anthracnose-twister disease complex is also widespread across the world in addition to waterlogging. Waterlogging is the major predisposing factor for anthracnose and other fungal diseases. However, studies on the combined stress impact on onions have been ignored. In the present review, we have presented an overview of the anthracnose-twister disease, the waterlogging effect on host physiology, host-pathogen interaction under waterlogging stress, and appropriate management strategies to mitigate the combined stress effects. Crucial soil and crop management strategies can help cope with the negative impact of concurrent stresses. Raised bed planting with drip irrigation, the use of plant bio-regulators along with nutrient management, and need-based fungicide sprays would be the most reliable and feasible management options. The most comprehensive solution to withstand combined stress impacts would be a genetic improvement of commercial onion cultivars.

## Introduction

**Figure 1:**
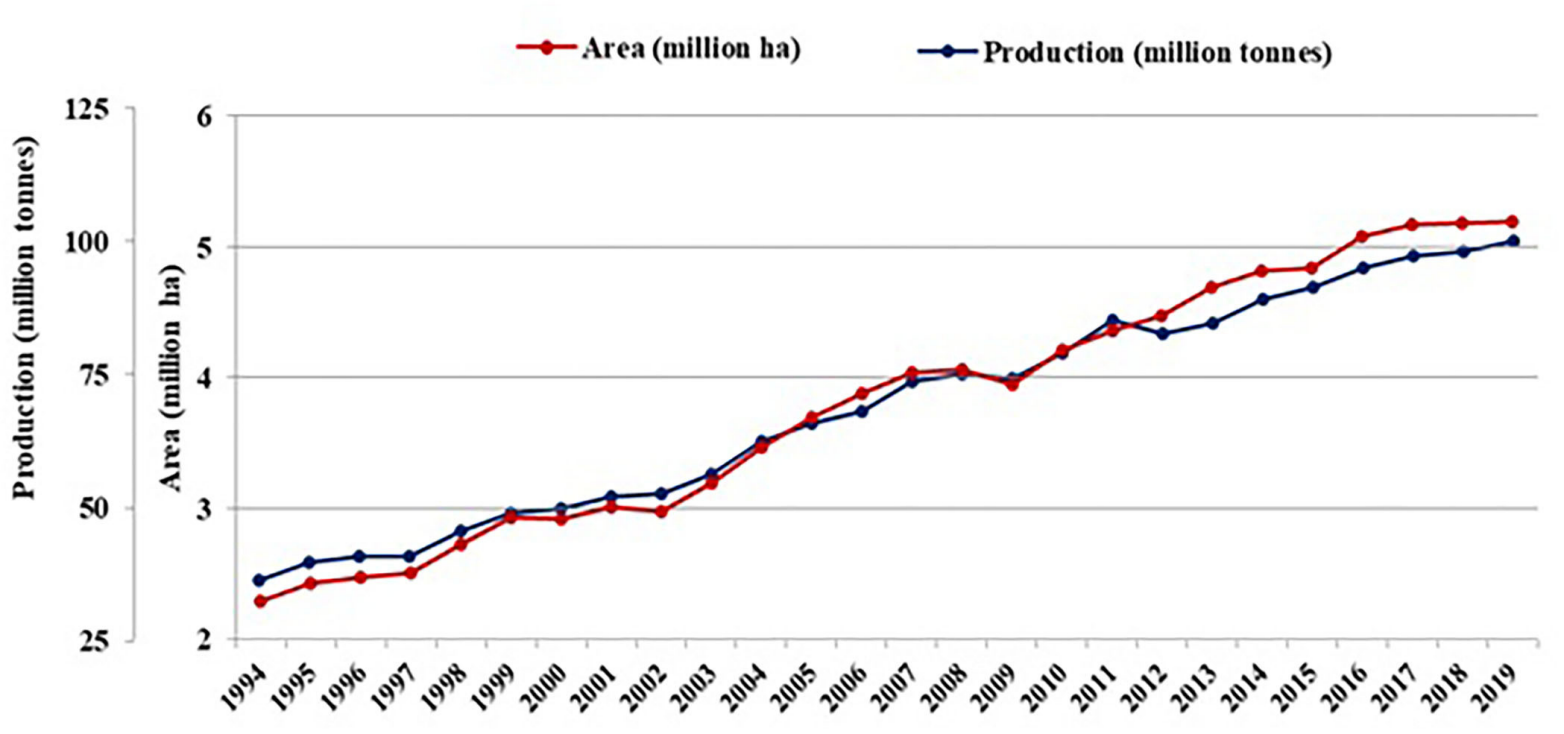
World status of onion on area harvested and production (Source: FAOSTAT, 2019).

Onions (*Allium cepa* L.), belonging to the family Alliaceae, are a major commodity in international trade due to their culinary and therapeutic uses. It has long been valued worldwide for its nutraceutical properties, including its anticancer, antidiabetic, antimicrobial, cardiovascular, and antioxidant effects. Onions are grown on more than 5.1 million hectares worldwide, yielding 99.9 million tons. Asia is a major contributor to the total production (64%), largely from China, followed by India (FAOSTAT, 2019). Despite the increased demand for onions in the international market, production is exhibiting a decreasing trend (Figure 1). The production, productivity, and innate nutritional potential of onions are immensely affected by the intervention of biotic and abiotic stresses. Diseases like Purple Blotch, Stemphylium Blight, the Anthracnose-Twister Complex, Fusarium Basal Rot, and insect pests such as thrips are the challenging biotic stresses affecting onion production. Among abiotic stresses, drought, waterlogging, extreme temperatures, salinity, and nutrient stress affect onion production; however, due to its shallow-rooted nature, it is extremely sensitive to waterlogging stress (Rao, 2016).

Further, owing to global climate change, there is a discernible change in the onset and withdrawal of the monsoon and erratic rainfall patterns, resulting in frequent and prolonged waterlogging events [Jackson and Colmer, 2005; Intergovernmental Panel on Climate Change (IPCC)., 2014]. These climatic aberrations encouraged abiotic stresses, which, in turn, affected biotic stresses in onions; the classic example is the co-occurrence of waterlogging and anthracnose-twister disease.

The first and foremost impact of waterlogging in a plant is anoxia and hypoxia within the roots and their effects on the shoots (Arduini et al., 2016; Herzog et al., 2016). In addition to changes in host physiology, waterlogging may attract various facultative parasites/saprophytes by increasing the host's susceptibility to pathogen attack and is thus believed to be one of the major predisposing factors in many plant disease infections (Hsu et al., 2013; Tacconi et al., 2015; Tosi et al., 2015; Urban et al., 2015; Sorrenti et al., 2019). Similarly, in onions, waterlogging may have a significant role in the development of debilitating anthracnose or twister disease. However, until today, the interference of waterlogging with anthracnose-twister development has gone unnoticed. Though the predictable losses due to the disease vary with its severity and crop growth stage, the pathogen *Colletotrichum* sp. has been reported to result in up to 80–100% onion bulb yield loss in several countries (Ebenebe, 1980a; Chawda and Rajasab, 1996; Alberto et al., 2001, 2019; Alberto, 2014). Moreover, this disease has forced the majority of farmers to abandon rainy-season onion cultivation in India, Sri Lanka, Indonesia, and many west African countries (Wiyono, 2007; Sikirou et al., 2011; Chowdappa et al., 2015; Herath et al., 2021).

India, the second-largest onion-growing country in the world, has a high demand for its onions in the global market due to their distinctive pungency and year-round availability. In India, onions are grown three times a year: during the rainy season *(kharif:* July to October) (20%), during the late rainy season (late *kharif* : October to January) (20%), and during the post-rainy season (*rabi*: December to April) (60%). Although the share of rainy season onions in the country's total production is relatively small (up to 20%), it has a significant impact on price stability, as it can ensure a continuous supply in the market in October, November, December, and January, when stored onions from the post-monsoon season *(rabi)* are not available in the market (Samra et al., 2006; Srinivas and Lawande, 2007; Gopal, 2015; Gedam et al., 2021). In the major onion-growing states of India, i.e., Maharashtra and Karnataka, anthracnose is a major obstacle during the rainy season. Intense rainfall leading to soil flooding/waterlogging coupled with anthracnose disease are the major factors affecting rainy-season onion production.

So far, the available literature focuses on the individual stress impacts (either waterlogging or anthracnose) on onions (Chawda and Rajasab, 1996; Alberto et al., 2001, 2019; Yiu et al., 2009; Alberto, 2014; Ghodke et al., 2018; Dubey et al., 2020, 2021; Gedam et al., 2021). Most of the time, multiple stresses coexist during the crop growth cycle. The impact of multiple stress co-occurrences and their interaction has not been studied. Therefore, through this review article, we highlighted an overview of anthracnose-twister disease, the effect of waterlogging stress on host physiology, host-pathogen interaction under waterlogging stress, and appropriate management strategies to mitigate the combined stress effects.

## Global and spatial distribution of anthracnose/twister

**Figure 2:**
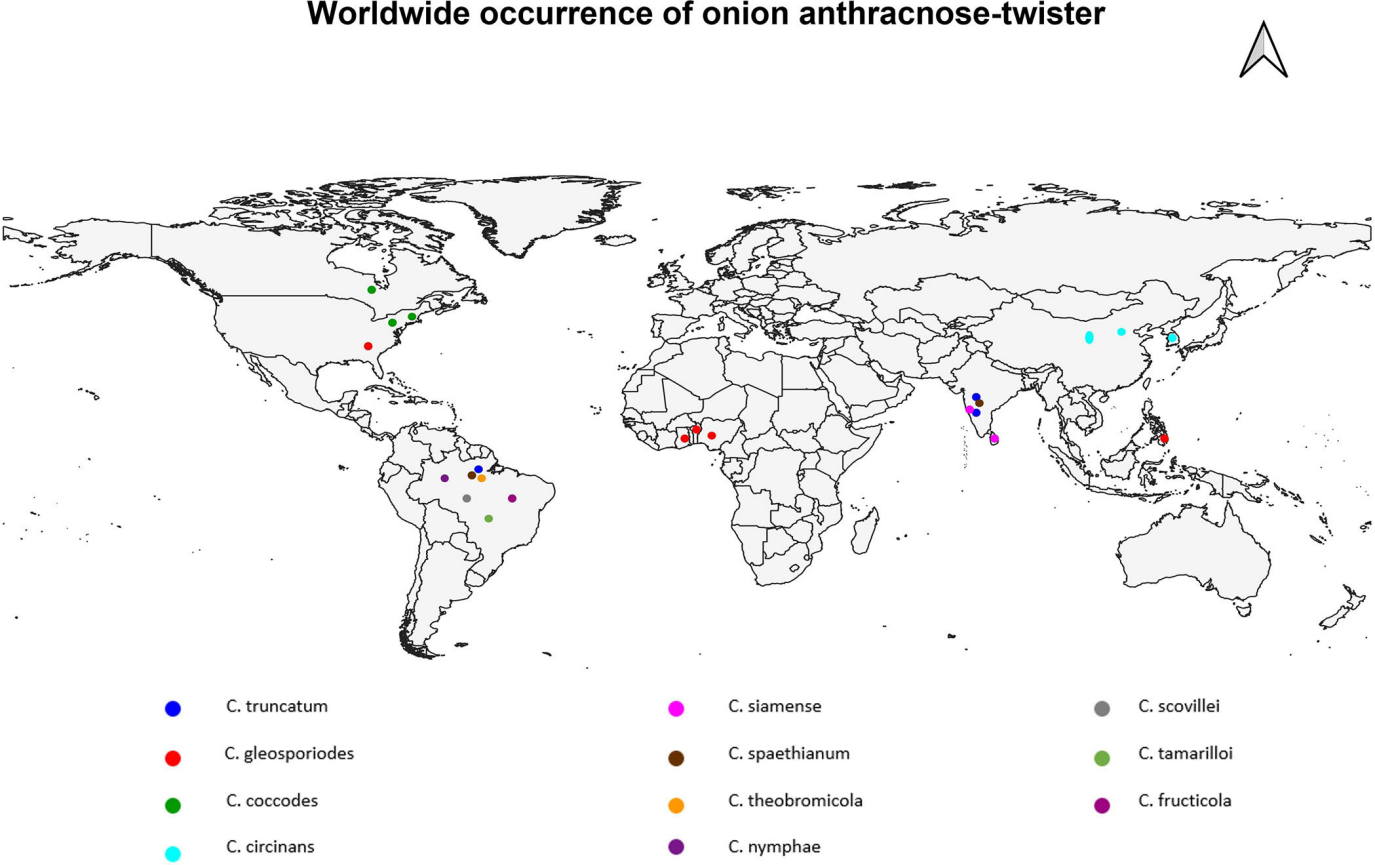
Global/spatial disease distribution of anthracnose.

Earlier studies discovered that this disease is of major concern wherever onions are grown during the rainy or monsoon season (Ebenebe, 1980a; Qadri and Srivastava, 1985; Qadri, 1988; Singh and Sinha, 1994; Galvan et al., 1997; Weeraratne, 1997; Kuruppu, 1999; Alberto et al., 2001, 2002; Wiyono, 2007; Kim et al., 2008; Nischwitz et al., 2008; Sikirou et al., 2011; Rodriguez-Salamanca et al., 2012; Baysal-Gurel et al., 2014; Chowdappa et al., 2015; Santana et al., 2015). Figure 2 shows that anthracnose-twister disease is widespread throughout the world and occurs most regularly in the tropics and subtropics.

## Taxonomic and molecular characterization of *Colletotrichum* sp. and genetic diversity of the pathogen

Conventional detection and characterization of Colletotrichum species were typically based on morphological characteristics, which were ineffective for distinguishing between Colletotrichum species. Hence, molecular characterization is much preferred and essential to examine genetic variation within and between species and populations. The internal transcribed spacer region (ITS) of ribosomal DNA has been normally used to separate Colletotrichum species (Freeman et al., 2000). However, it is not always appropriate (Damm et al., 2012). Therefore, molecular markers, actin (ACT), β-tubulin (TUB2), chitin synthase 1 (CHS-1), and glyceraldehyde-3-phosphate dehydrogenase (GAPDH) gene, have been used to identify the *Colletotrichum* species (Weir et al., 2012). Several studies of morphological and molecular characterization by earlier researchers were conducted on onions to determine the cause of the anthracnose-twister complex. Before 1997, *C. gloeosporioides* was known as the only pathogen responsible for onion anthracnose. Later, *C. gloeosporioides* and *Fusarium oxysporum* were reported to be the predominant microorganisms associated with the leaf twister disease (LTD) (Weeraratne, 1997; Kuruppu, 1999). Further, a detailed etiological study revealed that the onion anthracnose is a disease complex caused by the collective infection of *C. gloeosporioides, C. acutatum*, and *Gibberella moniliformis* (Alberto and Aquino, 2010). Among these, *C. gloeosporioides* and *C. acutatum* were reported to be responsible for leaf anthracnose, while *G. moniliformis* was responsible for abnormal neck elongation and leaf twisting, which might be due to excessive accumulation of phytohormone gibberellins in onions (Alberto and Aquino, 2010; Alberto, 2014). Several other *Colletotrichum* spp. such as *C. circinans* (Kim et al., 2008; Kiehr et al., 2012; Chen et al., 2022), *C. coccodes* (Rodriguez-Salamanca et al., 2012; Baysal-Gurel et al., 2014; Hay et al., 2016), *C. siamense, C. truncatum* (Chowdappa et al., 2015; Salunkhe et al., 2018a), *C. spaethianum* (Santana et al., 2015; Salunkhe et al., 2018b), and recently three members of *C. acutatum* species complex (*C. nymphaeae, C. scovillei*, and *C. tamarilloi*), and two members of *C. gloeosporioides* species complex (*C. fructicola, C. theobromicola*) (Lopes et al., 2021) have been reported in onion crop and their wild relatives. Thus, all earlier studies revealed that onion anthracnose is a disease complex predominantly caused by the *Colletotrichum* species complex and the association of *G. moniliformis*, which helps to increase symptom severity.

## Symptomatology

**Figure 3:**
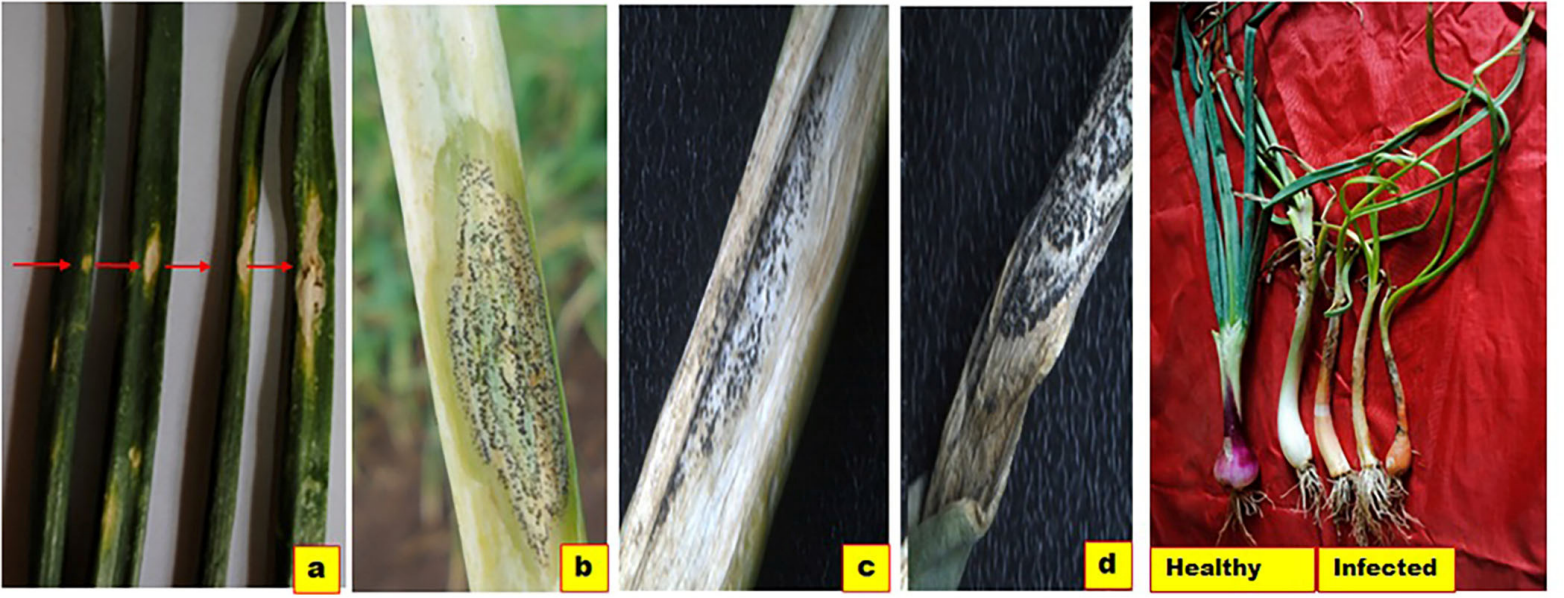
Symptoms of Anthracnose-Twister. **(a)** Initial white sunken lesions on leaves enlarges to form straw color patches surrounded by chlorotic margin. **(b)** Lesion advancement with formation of black color, fruiting bodies (acervuli). **(c)** Necrotic tissues covered with acervuli. **(d)** Leaf twisting and necrosis **(d)** seedling chlorosis, neck elongation and twisting with undersized bulbs. Red arrows indicate lesion enlargement.

This disease affects both the aboveground and underground parts of the onion. Typical anthracnose-affected plants showed leaf curling, twisting, chlorosis, necrotic leaves and sheath with black acervuli, and neck/pseudostem elongation followed by bulb rotting (Ebenebe, 1980a; Chawda and Rajasab, 1996; Weeraratne, 1997; Madushani et al., 2012; Chowdappa et al., 2015) (Figure 3). We observed that initially, small, whitish, water-soaked sunken lesions appeared on leaf blades and leaf sheaths. Further, the lesions became oval to elliptical and were surrounded by chlorotic margins, which turned into necrotic regions along the leaf axis. On necrotic tissues, prominent, black acervuli were formed in concentric rings. Later, the lesions became thin, brittle, and easily detached at the point of the lesion. Under high inoculum pressure, lesions can be seen on the leaf sheath near the soil, leading to bulb rotting. If the disease occurs at an early crop stage, there is no bulb formation, and it can cause complete crop loss. If it occurs at a later stage, even if there is bulb formation, the quality of the bulb deteriorates (Alberto et al., 2001, 2002; Wiyono, 2007; Nischwitz et al., 2008).

## Pathogen infection strategy

In nature, *Colletotrichum* spp. can overwinter in the form of acervuli/dormant mycelium on infected plant debris in the soil. With the onset of monsoon rains or sometimes off-season showers, resting structures enhance conidia formation (Ebenebe, 1980b), which establishes contact with the host for establishment. These conidia are usually water-borne, and quiescent infection is at its utmost during the wettest days of the cropping season (Denham and Waller, 1981; Fitzell and Peak, 1984; Darvas and Kotze, 1987). A detailed study of conidial behavior showed that since conidia were embedded in a mucilaginous mass of glycoproteins and proline-rich substances, they remained protected from desiccation and toxic plant metabolites (Gregory et al., 1959; Nicholson, 1992). Mucilage also has the self-inhibiting compound gloeosporone, which prevents conidial germination in thick suspensions (Lax et al., 1985). With rainfall and high humidity, mucilage absorbs water, and thus conidial concentrations become diluted.

Moreover, the liberation and dispersal of conidia take place through a splash and wash-off mechanism (Rajasab and Chawda, 1994). Lower leaves receive conidial inoculum with rain splashes; hence, the maximum infected area was observed on the lower leaves. Later, conidia were transported by wash-off mechanism, along with rain splashes, to the upper leaves and were released from the leaves, moved downward, and deposited over the neck and bulbs where anthracnose symptoms develop. Since the conidial inoculum is distributed in several loci, the disease initially appears in patches and is equally distributed throughout the field (Ebenebe, 1980b).

**Figure 4:**
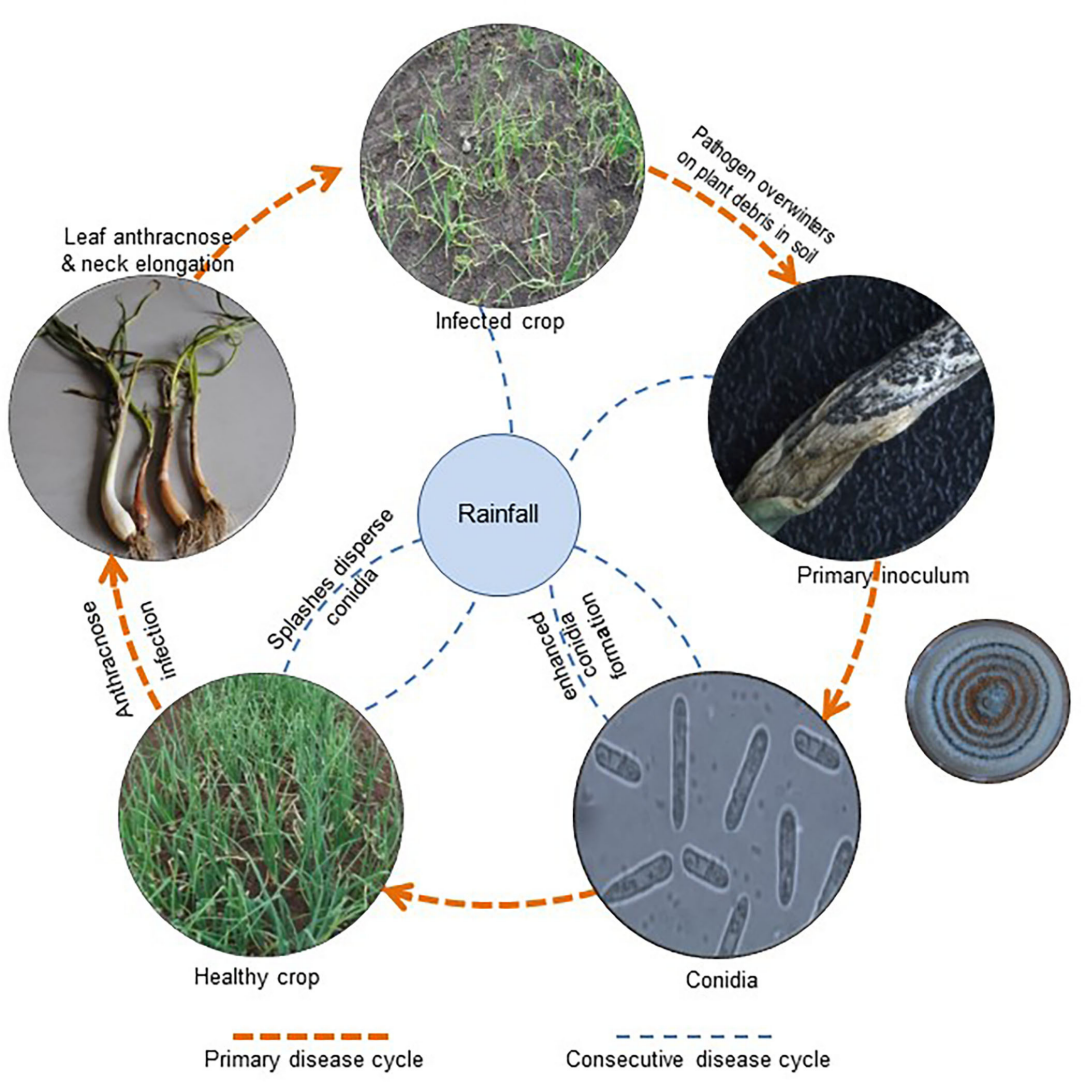
Disease cycle of *C. gloeosporiodes* on *Allium cepa*.

After reaching the infection site, conidia rapidly adhere to the aerial parts of the host plant (Nicholson, 1992, 1996; Mercure et al., 1994a,b) and apply intracellular colonization and subcuticular intramural colonization strategies for infection. Besides this, the fungus develops infection structures such as germ tubes, appressoria, intracellular hyphae, and secondary necrotrophic hyphae (Bailey et al., 1992). Pre- and post-penetration steps of infection, colonization, and anthracnose symptom appearance in onions could be completed within 120 h after incubation (Panday et al., 2012). Thus, the pathogen with a short life cycle (Figure 4) may trigger disease outbursts under a conducive environment and has the potential to destroy the crop in a very short span.

## Epidemic favoring climatic conditions

Studies regarding anthracnose favorable weather parameters reported that heavy rainfall, high relative humidity (nearly 100%), and moderate temperature (20–31°C) appear to be congenial for spore germination, pathogen establishment, and disease epidemics (Mordue, 1971; Weeraratne, 1997; Sharma and Kulshrestha, 2015). Similar studies in tomato and pepper also concluded that anthracnose severity was found to be associated with intense rainfall or overhead irrigation, which encourages conidial dispersal and provides leaf wetness favorable for pathogen infection and disease development (Dillard, 1989, 1992; Sanogo and Pennypacker, 1997). Interaction between temperature and relative humidity plays an important role in anthracnose symptom severity, and a combination of high (95 ± 5%) RH for ≥24 h and temperatures ≥25°C creates highly conducive conditions for anthracnose (Rodriguez-Salamanca et al., 2018). Thus, it is obvious that rainfall causing high relative humidity and prolonged leaf wetness are the primary contributors to disease formation and progression. Waterlogging stress and anthracnose co-occur during the onion crop cycle. Therefore, their co-occurrence might have a certain interaction that needs to be systematically investigated.

## Waterlogging stress physiology and its impact on anthracnose

**Figure 5:**
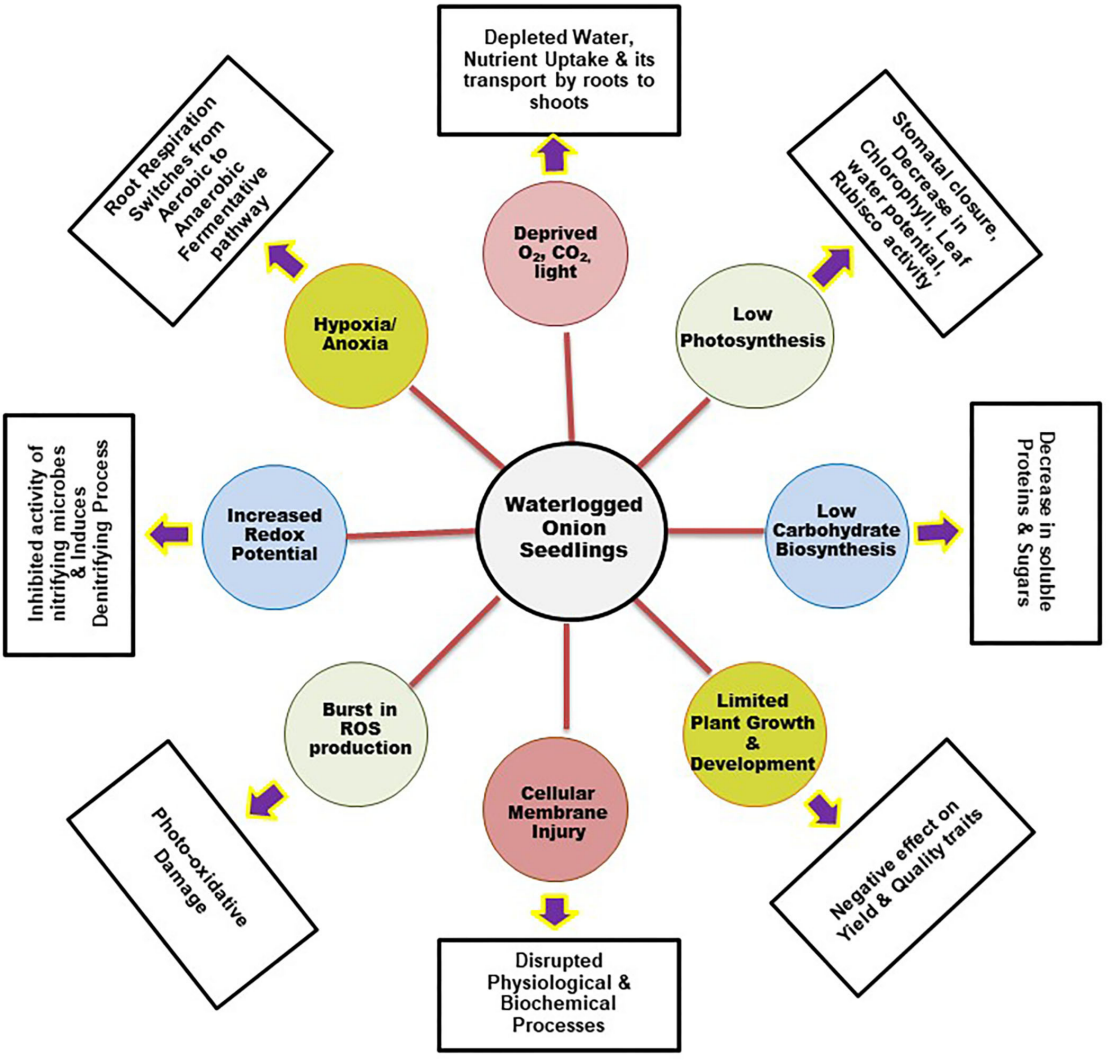
Physiological changes in host due to waterlogging stress.

Soil flooding/waterlogging is a widespread, unavoidable seasonal phenomenon that limits onion production globally. The damage due to waterlogging is severe in onion crops because of their shallow root systems, where maximum root penetration is about 75 cm, with higher root density only in the topsoil layer (Drinkwater and Janes, 1955). It adversely affects plant growth and development both in the nursery and in a standing crop and, consequently, the yield potential of onions (Ghodke et al., 2018). It also limits plant growth by interfering with various physiological and biochemical processes regulated by a network of genes and plant hormones (Figure 5). Its impact is complex and varies according to the variety, season, soil type, growth stage, extent, and rainfall duration.

Waterlogging stress leads to oxygen scarcity in the soil as it reduces soil aeration. Roots are the primary organ that experiences the damaging effects of flooding stress. Waterlogging stress often creates hypoxia (low oxygen) or anoxia (absence of oxygen) in the roots that limits photosynthetic oxygen production. Consequently, this affects the internal oxygen level and diffusion, which shifts the root respiration from aerobic to a low-ATP, anaerobic fermentation pathway. This induced ethanolic and lactic fermentation pathway *via* mitochondrial oxidative phosphorylation depletes the overall plant energy level. This energy crisis inhibits root growth and its functioning, leading to root mortality. Along with soil physicochemical properties (pH and redox potential) low oxygen level also affects the soil microbiota. Waterlogging lowers the concentration of oxidized nutrient elements (NO_3_^-^, SO_4_^2-^, Fe^3+^) and increases the abundance of reduced elements (Mn^2+^, Fe^2+^, H_2_S, NH_4_^+^). These elements accumulate in the root in addition to the internally synthesized ethylene and carbon dioxide that are highly toxic to plants (Greenway et al., 2006; Kreuzwieser and Rennenberg, 2014). It further induces oxidative stress in plants by stimulating a progressive reduction in soil oxygen levels. This causes a burst in the production of reactive oxygen species (hydrogen peroxide, superoxide, and hydroxyl radical), resulting in photo-oxidative damage that leads to cell membrane damage, enzyme deactivation, impaired photosynthesis, and other vital processes that interrupt the normal water and nutrient translocation toward the shoots (Herzog et al., 2016). Eventually, the overall plant growth is stunted due to energy limitation; under severe anaerobic conditions, the accumulation of toxic products further leads to high plant mortality.

Photosynthesis is the key physiological process that is severely affected due to waterlogging stress. The net carbon assimilation rate is hampered in many crops due to waterlogging stress (Vu and Yelenosky, 1991). The decrease in photosynthesis under hypoxia or anoxia is mainly due to stomatal and non-stomatal limitations. Reduced chlorophyll pigments, accumulation of carbohydrates, and alteration in Rubisco carboxylase/oxygenase and PEP carboxylase enzyme activities are associated with non-stomatal limitations (Iglesias et al., 2002). However, low stomatal conductance (Stomata closer) due to reduced root hydraulic conductivity is the stomatal limitation that impaired the water and nutrient absorption during the stress period. Plants with these limitations exhibited stunted shoot growth, declining leaf area, and premature leaf senescence, resulting in the yellowing of the leaves, wilting, and epinasty. In addition, the synthesis and signaling of plant hormones, photosynthesis, assimilation partitioning, and translocation are also negatively affected (Ferrer et al., 2005). These physiological impedances reduce crop yield (maize and okra) under waterlogged conditions (Vwioko et al., 2017). Nutrient deficiency is one of the major effects of waterlogging stress in plants, resulting in reduced photosynthesis and net carbon fixation, ultimately leading to a reduction in growth and yield (Bange et al., 2004) (Figure 5).

Waterlogging interferes with host physiology, pathogen biology, and plant-pathogen interaction and influences the incidence and severity of foliar diseases. Prolonged mild, wet weather and wet soil create high humidity within the crop canopy, which causes the pathogen to spread (Lawyer, 1985). Even though there have been several reports of the effects of leaf wetness or the amount and duration of rainfall on foliar diseases (Murray et al., 1990; Trapero-Casas and Kaiser, 1992), we have few reports regarding the effect of saturated soils on foliar diseases in the database. Generally, abiotic stress may influence plant-pathogen interaction positively or negatively, thus increasing or decreasing the disease severity (Chojak-Kozniewska et al., 2018). Seed-to-seedling transmission of bacterial blight (*Pseudomonas syringae* pv. *pisi*) in pea crops increases extensively as the soil approaches saturation (Roberts, 1992). Diseases of cereals and lupin crops are more severe in waterlogged soils (Belford et al., 1992). Waterlogging was found to increase the prevalence of banana vascular wilt (*F. oxysporum* f. sp. *cubense*), crown and root rot (*Phytophthora* spp.) of apple and raspberry damping off (*Pythium irregulare*) in beans, and verticillium wilt (*Verticillium dahlia*) in chili pepper (Duncan and Kennedy, 1989; Wilcox, 1993; Shivas et al., 1995; Aguilar et al., 2000; Sanogo et al., 2008; Li et al., 2015). Pathogens' ability to grow under anaerobic conditions in waterlogged soils contributes to disease severity (Rao and Li, 2003). The duration of waterlogging is an important component of an interaction study that can alter combined stress consequences. Waterlogging for more than 6 days stressed Cape gooseberry plants, making them more prone to wilt (*F. oxysporum* f. sp. *physali*.).

Onset and progression of onion anthracnose relies heavily on favorable environmental conditions such as., high humidity, leaf wetness, persistent precipitation and warm temperature (Mordue, 1971; Dillard, 1989, 1992; Sanogo and Pennypacker, 1997; Weeraratne, 1997; Sharma and Kulshrestha, 2015; Rodriguez-Salamanca et al., 2018). Abiotic stress usually increases vulnerability to hemibiotrophic or necrotrophic pathogens. However, it decreases susceptibility to biotrophic pathogens (Saijo and Loo, 2020). Since *C. gloeosporiodes* is a hemibiotrophic pathogen, waterlogging may increase the host's susceptibility to onion anthracnose. In addition to causing root anoxia, waterlogging raises canopy humidity, making the plant more susceptible to foliar diseases (Jolly et al., 2017).

**Figure 6:**
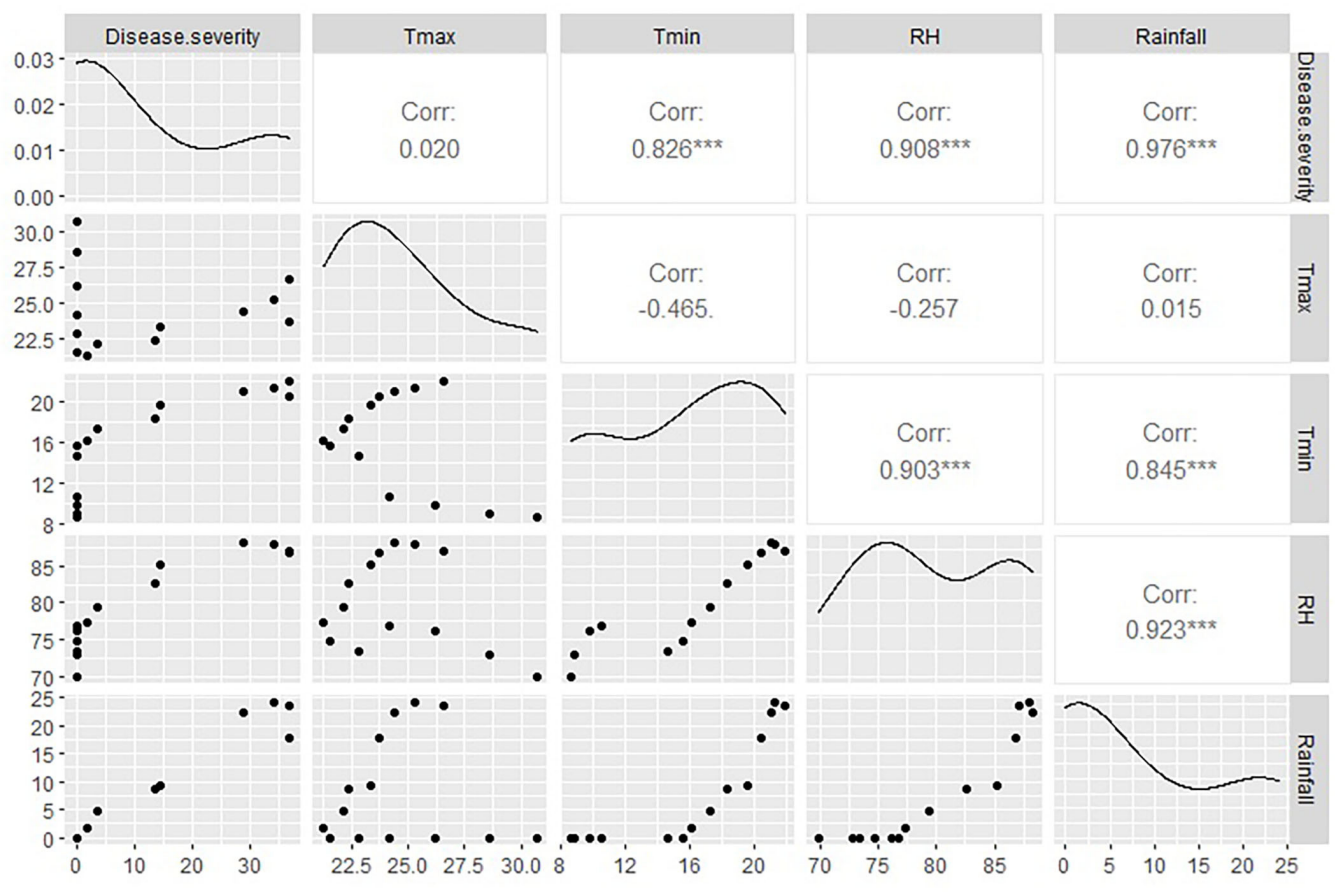
Anthracnose disease severity in relation to weather parameters (rainfall, humidity, temperature) during 2012-13 at ICAR-DOGR. ***Highly significant at < 0.001.

It is evident from the studies conducted during 2012–13 at the research station of ICAR-DOGR, Rajgurunagar, Pune, where the incidence of foliar diseases was monitored on 15 planting dates starting from June 15 to January 31, each at a 15-day interval. The peak incidence of anthracnose was during August and September, irrespective of the crop age/planting date. The highest significant positive correlation was recorded between disease severity and rainfall, i.e., 0.976 (p < 0.001). Even though several weather parameters are present, rainfall alone is able to play a significant role in anthracnose severity (Figure 6). During these months, high levels of soil saturation and frequent and long spells of water stagnation were observed in the field. These results support the fact that waterlogging aids in the development of anthracnose.

**Figure 7:**
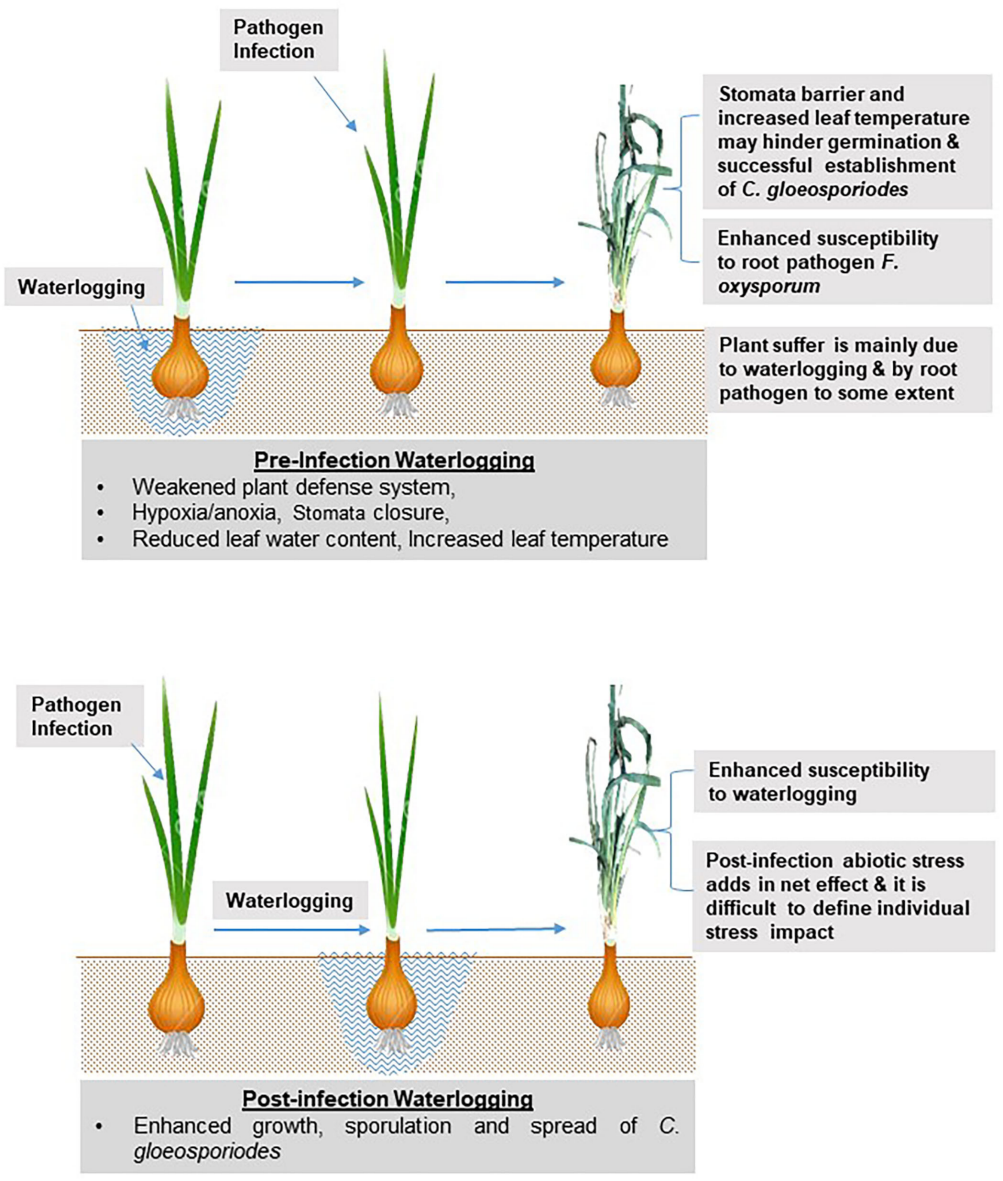
Effect of combined waterlogging and anthracnose/twister disease on onion (*Allium cepa*).

Generally, if abiotic stress happens before infection, it acts as predisposing stress, and predisposition implies an effect on the host rather than the pathogen; this also changes the host's resistance to susceptibility or, rarely, *vice versa* (Figure 7). In peas, pre-infection waterlogging reduced black spot (*Mycosphaerella pinodes)* severity, whereas post-infection waterlogging increased disease severity and caused a greater reduction in plant growth (McDonald and Dean, 1996). Pre-infection waterlogging might cause certain changes in host plants that hamper the pathogen establishment, and this might be the possible reason for reduced disease severity. Usually, there is a marked reduction in leaf water content (free water and relative water content) in waterlogged plants (Kuai et al., 2014; An et al., 2016; Zhang et al., 2019). The leaf water content is always associated with the water uptake capacity of the root, leaf transpiration, and leaf anatomy structure, which can alter due to waterlogging stress—the leaf temperature of waterlogged plants increases due to reduced transpiration rate and gas exchange.

Additionally, many plants close their stomata in response to waterlogging. Thus, in such situations, initial establishment and host penetration by the foliar pathogens may become difficult, reducing the possibility of anthracnose infection. However, under this condition, a possible increase in root infection may give a twister appearance to onion (Shivas et al., 1995; Alberto and Aquino, 2010; Alberto, 2014). In contrast, if the foliar infection by *C. gloeosporiodes* has already been established in the host and later the host is exposed to waterlogging, chances of enhanced growth, sporulation, and the spread of foliar pathogen may increase (Figure 7).

Moreover, nutritional imbalance due to waterlogging causes leaf chlorosis, senescence, wilting, necrosis, and stunted plant growth, which may affect the susceptibility of a plant to disease and increase the severity of infection (Graham, 1983; Yiu et al., 2008). The physiological response of plants to waterlogging may also make them more susceptible to disease. Increased ethylene levels in the shoots of waterlogged plants may be responsible for chlorosis and senescence (Smith, 1987). Stem/neck elongation is an escape strategy by short-term waterlogged plants (Zhang et al., 2021). In onions, abnormal neck elongation and leaf twisting are supposed to be due to excessive accumulation of gibberellins produced by secondary infection of *G. moniliformis* (Alberto and Aquino, 2010; Alberto, 2014). Nevertheless, the role of waterlogging in onion neck elongation is still unclear, and studies in this line may provide a clear picture.

In several cases, exposure of plants to abiotic stress weakens disease resistance, while pathogen infections often enhance abiotic stress responses (Atkinson and Urwin, 2012). Plants' response to combined stress is a more complex phenomenon than individual stress response and warrants an understanding of the crop-specific response. Thus, in addition to other factors such as a virulent pathogen, an initial amount of inoculum, susceptible hosts, a congenial environment, and crop management practices (planting method and plant density), waterlogging has a role in disease development that needs to be systemically studied. Knowledge of pre- and post-infectional waterlogging impact on onion anthracnose development will help combat combined stress.

## Concurrent waterlogging and anthracnose-twister management strategies

Adopting site-specific soil and crop management practices can improve soil drainage and trigger plant resistance, respectively, and may help mitigate the negative impact of combined stress. Although specific research work in this direction is yet to be carried out, possible management options that might help to combat the concurrent stress in onion are discussed below.

## Raised bed with drip irrigation

Raised bed planting should be followed for planting rainy season onions to minimize the damage due to water stagnation and anthracnose (Govaerts et al., 2007; Gadge and Lawande, 2012; Laxman et al., 2020). In raised beds, as the top 15 cm of the soil layer remained unsaturated, it decreased the waterlogging effect and improved soil drainage by creating a favorable environment for root growth and its proper functioning (Bakker et al., 2005; Blessitt, 2007; Velmurugan et al., 2016). Further, bioaerosols (small water droplets with conidia masses) were easily disseminated, and disease incidence might increase with overhead irrigation (Raniere and Crossan, 1959). Hence, the adoption of drip irrigation was found to be more effective in avoiding water stagnation in the root zone and minimizing disease incidence (Tripathi et al., 2017). Thus, a raised bed with drip irrigation could be an efficient technology for mitigating waterlogging and anthracnose stress in rainy onions.

## Enhanced crop vigor

Crop vigor during the initial growth stages can be essential for waterlogging and disease tolerance (Sundgren et al., 2018). Seed priming is the most effective method for improving seedling establishment and early crop vigor (Taylor et al., 1998). Various crop seed priming agents have been explored for abiotic and biotic stress tolerance. In onions, vermicompost seed priming enhanced germination, and healthy plants withstood drought, salinity, and temperature stresses (Muhie et al., 2020). This was due to the improved activities of enzymes such as catalase, superoxide dismutase, and ascorbate peroxidase. Priming with hydrogen peroxide (H_2_O_2_) enhanced waterlogging tolerance in soybean seedlings (Andrade et al., 2018), which can be explored in onions. Nevertheless, more research is needed to determine the impact of vigor-stimulating compounds and their use for concurrent stress alleviation in onions.

Further, there are reports of managing early-stage crop stress with emerging plasma technology (Song et al., 2020). Limited *in vitro* studies regarding the induction of crop tolerance to pathogen stress (Jiang et al., 2014; Ochi et al., 2017), water deficit (Ling et al., 2015; Guo et al., 2017; Feng et al., 2018), salinity (Iranbakhsh et al., 2018), and oxidative stress (Bubler et al., 2015; Iranbakhsh et al., 2017) by plasma technology have been documented. However, validation of *in vitro* results through field experiments is needed to confirm the use of plasma technology in tackling the mentioned concurrent stress in onions.

## Nutrient management

Soil nutrient losses due to waterlogging can cause significant impact on plant heath. Proper nutrient management is needed to mitigate the harmful effects of waterlogging and attain better crop performance (Noreen et al., 2018). In waterlogged conditions, soil nitrogen (N) is lost due to leaching, resulting in reduced uptake by plants and N deficiency in both plants and soil (Nguyen et al., 2018). Early crop vigor may be linked to enhanced nitrogen uptake (Sundgren et al., 2018). Additionally, plant growth and development can be improved with the application of slow-release N fertilizers, as they release nitrogen over a prolonged period during crop growth and make it available as per crop demand (Shaviv, 2001; Dinnes et al., 2002; Lubkowski and Grzmil, 2007; Varadachari and Goertz, 2010; Trenkel, 2021). Though there are several reports on the advantages and disadvantages of the application of N fertilizer during or immediately after waterlogging on crop yield (Swarup and Sharma, 1993; Najeeb et al., 2015; Mondal et al., 2018), the efficiency of the same in onions needs to be studied in detail.

Similarly, potassium is an essential nutrient in various physiological and biochemical plant mechanisms and can trigger abiotic and biotic stress tolerance/resistance in plants. Mitigating K^+^ loss due to hypoxia or anoxia stress is an important strategy for building up waterlogging resistance in plants (Mancuso and Marras, 2006; Pang et al., 2006; Mugnai et al., 2011; Teakle et al., 2013). Potassium supplementation under waterlogging stress enhances plant growth, photosynthetic pigments, rate of photosynthesis, and plant nutrient uptake (Ashraf et al., 2011). However, the efficiency of exogenous potassium application in alleviating the negative effects of waterlogging and disease incidence is variable depending on the source and amount of K, application time, and plant and pathogen species (Prabhu et al., 2007). Therefore, the role of potassium supplementation in mitigating waterlogging and anthracnose stress in onions is still unclear and needs focused research.

## Use of plant growth regulators (biostimulants)

Plant bio-regulators may alleviate the harmful effects of waterlogging stress if applied at the correct growth stage (Nguyen et al., 2018; Ren et al., 2018; Wu et al., 2018). Foliar application of spermidine and spermine in Welsh onions (*Allium fistulosum*) stimulated biochemical and physiological adaptations during waterlogged stress (Yiu et al., 2009). However, evidence suggests an efficient use of plant bio-regulators such as 1-naphthaleneacetic acid (1-NAA), cytokinin, 6-benzyl adenine (6-BA), 5-aminolevulinic acid (ALA), glycine betaine (GB), abscisic acid (ABA), gibberellic acid, ethylene, and melatonin in enhancing waterlogging tolerance in various crops (Pandey et al., 2002; Pang et al., 2007; An et al., 2016; Ren et al., 2016, 2018; Kim et al., 2018a,b; Rasheed et al., 2018; Zhang et al., 2019). Adequate studies in this field need to be carried out in onions to depict the exact hormonal and enzymatic cross-talk, metabolic pathways, and physiological, morphological, and anatomical adaptations governing waterlogging tolerance. Further, the efficiency of growth regulators activating the onion defense mechanism under waterlogged conditions needs to be explored.

As nutrients can build up plant immunity and growth regulators help in the recovery of physiological injury, integrated application of nutrient and growth regulators at the proper crop stage could be a worthwhile strategy in ameliorating waterlogging and anthracnose stress in onion.

## Biological management

The damage caused by waterlogging stress can be managed to some extent by treating plants with ACC deaminase–producing PGPR (Barnawal et al., 2012; Li et al., 2013). Pseudomonas putida UW4, Bradyrhizobium, and Enterobacter cloacae are some ACC deaminase producers that reduce the damage caused by waterlogging (Grichko and Glick, 2001; Fougnies et al., 2007; Grover et al., 2011). However, all these studies were conducted on other crops and needed to be investigated in onion. Moreover, the anthracnose management potential of these PGPR bacteria needs to be explored.

## Use of fungicides

Periodic, need-based fungicide sprays are one of the promising methods for mitigating biotic stress in plants. The selection of fungicide, timing of application, and spray frequency are critical for satisfactory anthracnose management. Predictive models based on rainfall and humidity will provide the necessary directions for fungicide application. Generally, copper fungicides, dithiocarbamates, benzimidazoles, and triazoles are recommended for anthracnose management (Waller, 1992). New-generation chemicals like strobilurins are also used for anthracnose management. Although the application of fungicides in managing onion anthracnose is widely popular, it is poorly documented. The disease can be treated with protective fungicides, namely mancozeb or chlorothalonil. Spraying 10 or 15 g/20 liters of Carbendazim at 5- or 10-day intervals effectively controlled anthracnose in onions (Anonymous., 1996). Besides this, strobilurins (Quadris and Cabrio) are also effective when used in rotation with a protectant to prevent fungicide resistance (Miller, 2013).

Further, there are a few reports of *C. gloeosporiodes* resistance to benzimidazole in papaya and mango anthracnose (Spalding, 1982; Astua et al., 1994; Barquero and Arauz, 1996). To avoid fungicidal resistance among the pathogen population, benomyl was included in the spray schedule (Jimenez et al., 1989). Triazoles can perform a dual role as fungicides and plant growth regulators to defend plants from various stresses (Leul and Zhou, 1998, 1999; Habibzadeh et al., 2013; Rademacher, 2015). It was found that Paclobutrazol (PBZ) pre-treatment enhanced antioxidants and antioxidative enzymes in sweet potato under flooding stress (Lin et al., 2006). Paclobutrazol (PBZ) application improved waterlogging stress tolerance in Welsh onions (Yiu et al., 2008). PBZ and carbendazim can perform well and show the lowest disease severity. Additionally, PBZ alone and with benomyl showed the shortest neck elongation (Perez and Alberto, 2020). Although few triazoles are already in use in managing anthracnose in onion, their role in the mitigation of both waterlogging and disease stress needs to be validated.

## Crop improvement

A cost-effective, eco-friendly, and widely adopted approach for diminishing the losses caused by waterlogging and disease is to introduce waterlogging as well as disease tolerance/resistance into existing plant varieties through breeding approaches (Zhou, 2010; Tewari and Mishra, 2018; Wani et al., 2018; Gedam et al., 2021). Preliminary screening of onion accessions against waterlogging and anthracnose was carried out at ICAR-DOGR. Among the available accessions, red onion line 1666 and DOGR Hybrid-50 were moderately resistant to both stresses (Directorate of Onion Garlic Research, 2015, 2017). Nevertheless, the tolerance to waterlogging is a complicated characteristic that is governed by several different mechanisms, such as aerenchyma formation in roots (Zhang et al., 2015; Luan et al., 2018; Pujol and Wissuwa, 2018), tolerance to secondary metabolites (Pang et al., 2006), ion toxicities (Huang et al., 2018), the maintenance of membrane potential (Gill et al., 2018), and control of ROS production under waterlogging stress, with many QTL reported to be controlling these characteristics (Li et al., 2008; Zhou, 2011; Zhang et al., 2017; Gill et al., 2018; Huang et al., 2018). Recently, omics technologies like transcriptomics, proteomics, genomics, and metagenomics have created new opportunities in crop improvement research. These technologies help us identify the key genes, traits, and metabolic pathways associated with stress tolerance in onion crops (Ghodke et al., 2020; Khandagale et al., 2020). This information will open a new avenue that will assist in developing tolerant onion varieties. Similarly, the anthracnose resistance breeding program in onions has hardly been carried out. Detecting the resistance source is a crucial step for successful disease resistance breeding. Up till today, very little research in this direction has been conducted. As per existing data, limited resistant sources are available on *Allium cepa*; therefore, introducing resistance genes from wild relatives will be a better option. Previous researchers found that anthracnose resistance was polygenic and could be controlled by several genes (Melo and Costa, 1983; Galvan et al., 1997). Currently, no fully resistant cultivars or wild relatives against anthracnose are available.

During pathogenesis, the pathogen may secrete enzymes that could cause rapid hydrolysis of total sugars in the host tissues, resulting in decreased resistance (Jayapal and Mahadevan, 1968). Low levels of total phenols in leaves might be the reason for decreased resistance (Arora and Wagle, 1985; Bashan, 1986; Mishra et al., 2009; Alberto, 2014). Further, cultivars with high levels of protein, sugar, and phenols may show increased resistance (Alberto et al., 2002). The success of a breeding program relies on the identification of genes and related markers for various tolerance mechanisms, which enable breeders to identify tolerant or resistant genes. Since the major part of the onion growing region is assumed to be strongly exposed to waterlogging and anthracnose stress during *Kharif* cultivation, emphasis should be given to the large-scale screening of resistance sources for both stresses and their utilization for the development of stress-tolerant varieties.

## Conclusion and future prospects

Knowing and understanding abiotic (waterlogging) and biotic (anthracnose-twister) stress impact on rainy season onion performance is critical. Although research has pointed to individual stresses, concurrent stress possibilities should not be overlooked. Even if humidity, rainfall, temperature, and prolonged leaf wetness are key drivers for anthracnose outbreaks, waterlogging interactions are likely to significantly affect disease severity. Waterlogging impact may be either positive or negative and depends on the sequence of the stress events that should be critically analyzed. The scope of critical crop management options for enhancing host resistance should be widely explored. Exogenous application of several plant growth regulators (PGR) could be considered one of the leading methods for enhancing stress tolerance in onions. Together, foliar nutrient and growth regulator applications provide opportunities for future research. Genetic enhancement could be achieved through genetic engineering and modern molecular breeding platforms. Thus, significant attempts are required to breed onion cultivars with enhanced tolerance to stress combinations.

## Author contributions

VS and SG conceived idea, designed the study, and contributed to anthracnose related portion of the manuscript. PG, BG, AP, and RK contributed to waterlogging part of the manuscript. VS, AP, BG, and RK contributed in analysis and visualizations. VS, PG, AP, BG, and SG wrote the manuscript. All authors played role in revising manuscript and approved the final version.
